# The hibernating South American marsupial, *Dromiciops gliroides,* displays torpor-sensitive microRNA expression patterns

**DOI:** 10.1038/srep24627

**Published:** 2016-04-19

**Authors:** Hanane Hadj-Moussa, Jason A. Moggridge, Bryan E. Luu, Julian F. Quintero-Galvis, Juan Diego Gaitán-Espitia, Roberto F. Nespolo, Kenneth B. Storey

**Affiliations:** 1 Department of Biology and Institute of Biochemistry, Carleton University, 1125 Colonel By Drive, Ottawa, Ontario K1S 5B6, Canada; 2 Instituto de Ciencias Ambientales y Evolutivas, Facultad de Ciencias, Universidad Austral de Chile, Campus Isla Teja, Valdivia, Chile; 3 CSIRO Oceans & Atmosphere, GPO Box 1538, Hobart 7001, TAS, Australia

## Abstract

When faced with adverse environmental conditions, the marsupial *Dromiciops gliroides* uses either daily or seasonal torpor to support survival and is the only known hibernating mammal in South America. As the sole living representative of the ancient Order Microbiotheria, this species can provide crucial information about the evolutionary origins and biochemical mechanisms of hibernation. Hibernation is a complex energy-saving strategy that involves changes in gene expression that are elicited in part by microRNAs. To better elucidate the role of microRNAs in orchestrating hypometabolism, a modified stem-loop technique and quantitative PCR were used to characterize the relative expression levels of 85 microRNAs in liver and skeletal muscle of control and torpid *D. gliroides*. Thirty-nine microRNAs were differentially regulated during torpor; of these, 35 were downregulated in liver and 11 were differentially expressed in skeletal muscle. Bioinformatic analysis predicted that the downregulated liver microRNAs were associated with activation of MAPK, PI3K-Akt and mTOR pathways, suggesting their importance in facilitating marsupial torpor. In skeletal muscle, hibernation-responsive microRNAs were predicted to regulate focal adhesion, ErbB, and mTOR pathways, indicating a promotion of muscle maintenance mechanisms. These tissue-specific responses suggest that microRNAs regulate key molecular pathways that facilitate hibernation, thermoregulation, and prevention of muscle disuse atrophy.

To survive in challenging environments, many animals employ metabolic rate depression to strongly reduce their energy needs and extend the time that they can live using only internal fuel reserves. Survival strategies including daily torpor, hibernation, estivation, anaerobiosis, and freeze tolerance all make use of hypometabolism^1^,^2^. Many mammalian species use hibernation to endure seasonal periods of cold exposure and food scarcity; indeed, hibernation has been identified in selected species from eleven different mammalian orders including, monotremes, marsupials, rodents, bats and others^3^,^4^. Hibernation is an energy-saving strategy that involves a seasonal retreat into cycles of prolonged torpor where metabolic rate and core body temperatures are depressed^5^,^6^,^7^. Hibernators with small body masses can save 40–60% of their total daily energy needs by maintaining a decreased core temperature, compared with the high thermoregulatory costs of continuous endothermy^8^.

The temperate rainforests of Chile are home to the only known South American hibernator and the last living representative of the Microbiotheria order, the monito del monte (*Dromiciops gliroides*)^8^,^9^. This cold-adapted marsupial uses both daily torpor and multi-day hibernation as an opportunistic response to stress, where the duration and depth of torpor bouts are proportional to the intensity of cold exposure and food scarcity^8^,^10^. Bioenergetic studies have described the physiological features of *D. gliroides* hibernation; for example, when ambient temperature decreased to 12.5 °C, deep torpor bouts were found to last for five days and during this period core body temperatures fell to near ambient levels, metabolic rate dropped to 1–5% of the euthermic rate, and respiratory frequency fell from an average of 370 to 2 breaths per min with extended periods of apnea^6^,^8^,^11^. Throughout the year, when faced with less extreme environmental conditions, the nocturnal *D. gliroides* employs a different strategy, retreating daily into shallow torpor to manage energy expenditure and mass balance^11^ During these periods of shallow torpor, body temperature decreases to 11–28 °C for a few hours, and metabolism is suppressed to 10–60% of basal rate^8^,^11^.

A molecular hallmark of torpor is the reprioritization of ATP consumption through the suppression of energetically costly cellular metabolic processes such as gene transcription and translation. Yet, despite the observed global suppression of metabolism, selected genes that facilitate the transition between torpor/arousal states, promote the torpid state, or enhance pro-survival pathways have been found to be overexpressed during hibernation^12^. Apart from these metabolic changes that facilitate hypometabolism in all organs, selected organs can have specific roles to play during torpor/arousal. For example, skeletal muscle is crucial for arousal from torpor/hibernation since it provides shivering thermogenesis to help rewarm the animal. Therefore, it is important for *D. gliroides* to maintain muscle mass and function despite prolonged inactivity and, indeed, other mammalian hibernators show little or no sign of disuse atrophy during hibernation^13^,^14^. In eutherian mammals, thermogenesis during arousal also strongly depends on the action of brown adipose tissue but the presence of this heat generating organ has yet to be confirmed in marsupials. Detecting the occurrence of brown adipose mediated adaptive non-shivering thermogenesis in marsupials has yielded controversial results, where thermogenic responses were found in some marsupials^15^,^16^,^17^,^18^, while no such findings were observed in others^19^. Moreover, evidence of the morphological and molecular characteristics of brown adipose in marsupials are inconclusive^17^,^20^. Although orthologues of the three uncoupling proteins, evolutionary markers for brown fat, have been identified in a few marsupial genomes^21^,^22^, upon characterization it was found that they may not be contributing to classical non-shivering thermogenesis, and may have evolved different roles that have yet to be elucidated^23^. Therefore, if marsupials indeed lack brown adipose they must have evolved an alternative heat generating mechanism such as changes to liver metabolism that may be contributing to this function. Villarin *et al.*^24^ reported that sustained cold exposure elevated liver oxidative capacity in *Monodelphis domestica* (the North American opossum); liver mass increased by ~50% and liver mitochondrial volume rose ~20%^24^. These changes could account for nearly half of the increase in animal oxygen consumption at low temperatures suggesting that the liver has the potential to contribute significantly to the thermogenic efforts for marsupial life in the cold. Hence, these results, along with other studies, suggest that liver metabolic processes are actively modified to contribute to the heat production necessary for marsupial survival throughout torpor/hibernation^5^,^18^,^25^. Despite the differences in thermogenic mechanisms, normothermic body temperatures, and basal metabolic rates between marsupials and eutherian mammals, there appears to be no significant difference between heat production rates, suggesting that marsupials are able to warm-up as fast as eutherians despite a potential absence of brown adipose tissue^26^.

A complex balance of gene activation and suppression is needed to achieve hypometabolism and this can be accomplished by various molecular mechanisms, including reversible protein phosphorylation of transcription and translation factors, the use of epigenetic mechanisms to directly modify DNA and histone proteins, as well as post-transcriptional controls on mRNA through the action of microRNAs (miRNAs)^2^. MiRNAs are proving to be master regulators of virtually all cell functions with broad controls over many activities including the cell cycle, signal transduction pathways, and energy metabolism, among others^27^,^28^. While this large group of highly-conserved, short, non-coding RNA transcripts (~22 nt) comprises just 1–2% of the number of protein-coding genes, recent computational predictions indicate that they target more than 60% of protein-coding genes in humans, and influence almost every aspect of biological function^29^,^30^. MiRNAs are initially transcribed mostly by RNA polymerase II into long (~70 nt) secondary hairpin structures known as pri-miRNAs. The 5′ and 3′ ends are cleaved by the enzymes Drosha and DCGR8 to form a pre-miRNA, which is subsequently exported to the cytoplasm. At this stage, the stem-loop is cleaved by Dicer to create a ds-miRNA duplex and, from this, the guide strand is then incorporated into the miRNA-induced silencing complex (miRISC)^31^. The 5′ seed region of the guide strand, involving nucleotides two to eight, directs RISC activity towards target mRNAs with complementary motifs in their 3′UTR^32^. The brevity of the seed region is what allows miRNAs to exert their broad controls, wherein a single miRNA can regulate multiple mRNA transcripts, and one mRNA transcript may be subject to regulation by multiple miRNAs^31^. Once bound, the degree of complementarity between the miRNA and mRNA transcript dictates the fate of the mRNA transcript, providing another level of control. Imperfect complementarity causes mRNA transcripts to be sequestered to cytoplasmic loci such as P-bodies and stress granules, where they are subject to translational repression; in contrast, perfect complementarity triggers mRNA degradation by Argonaute endonucleases^31^.

A growing number of studies by our lab, and others, have demonstrated the essential role of miRNA differential expression in modulating global gene suppression and coordinating the up-regulation of selected genes in response to torpor, estivation, anaerobiosis, and freezing^27^,^33^,^34^,^35^,^36^,^37^,^38^,^39^,^40^,^41^. To our knowledge, the present study is the first to investigate miRNA expression in relation to torpor in a marsupial species. Eighty-five known miRNAs were quantified in the liver and skeletal muscle of control versus torpid *D. gliroides* using RT-qPCR. Genome alignments confirmed that the miRNAs analyzed were highly conserved as compared with other vertebrates. MiRNA expression was found to be differentially regulated in a tissue-specific manner, with significant suppression of many miRNA species observed in liver and the differential regulation of a subset of miRNAs in skeletal muscle. Bioinformatic target enrichment analyses suggested that the differentially expressed miRNAs may play a crucial role in facilitating hibernation and thermoregulation through the regulation of MAPK, mTOR, and PI3K-Akt signalling in liver. The skeletal muscle-specific miRNA expression profile was predicted to regulate the maintenance of muscle mass and function through the targeting and induction of the focal adhesion, ErbB, and mTOR signalling pathways during marsupial torpor. Understanding the role that miRNAs play as central regulators of metabolic reorganization will provide valuable insights into the molecular underpinnings of adaptation to environmental stress.

## Results

### Differential expression of miRNA in liver and skeletal muscle during torpor

**Figure 1:**
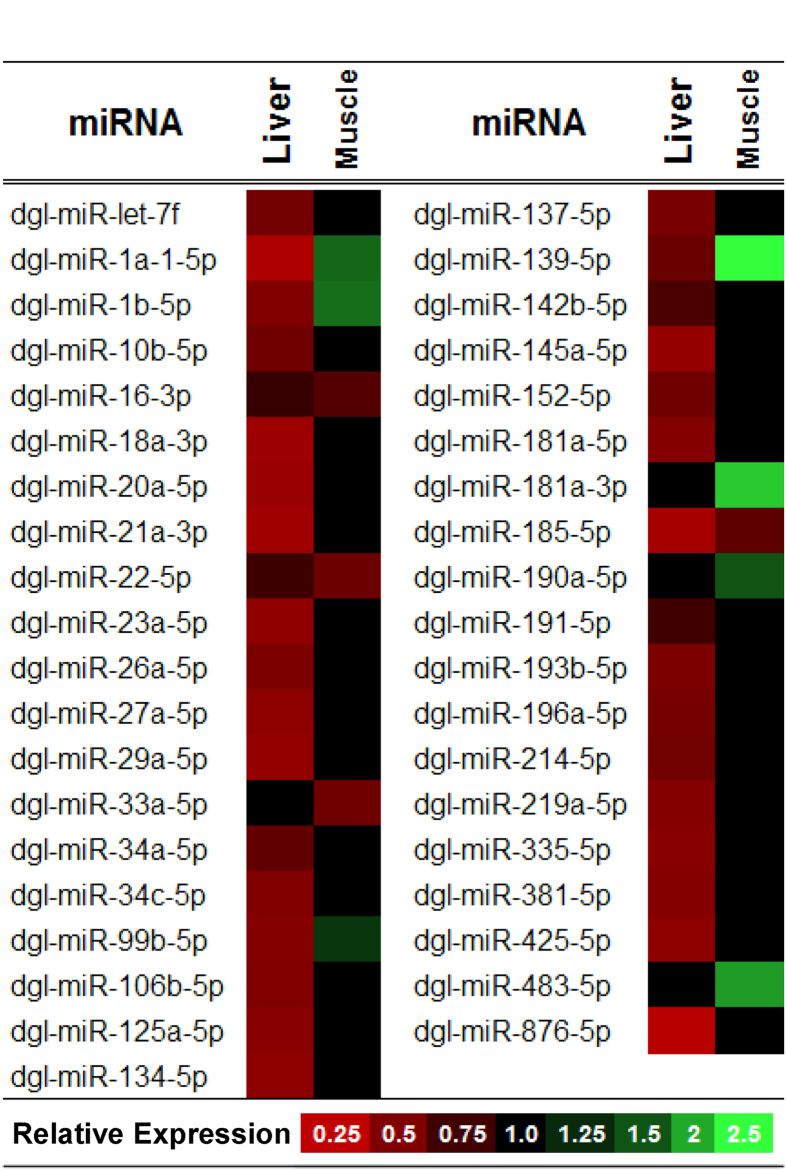
Heat map showing torpor-induced changes in the relative expression of 35 miRNAs in liver and 11 miRNAs in skeletal muscle of the marsupial *D. gliroides*. MicroRNA relative expression was evaluated by RT-qPCR of reverse-transcribed, polyadenylated transcripts. Data represent means of n = 4 biological replicates from different animals. Relative expression of genes was calculated by standardizing against *U6 snRNA* expression. Control values were adjusted to 1 and the torpid values were expressed relative to the controls. The reported miRNA expression level changes in torpid individuals were all statistically significant from the corresponding control; statistical testing used the Student’s *t-*test where *p* < 0.05. The legend provides a visual reference for the colour gradient used. Different shades of red represent significant downregulation of miRNA in the torpid state versus control; increasing redness signifies greater relative downregulation during torpor. Black represents no significant changes. Increasing greenness represents greater upregulation of miRNA during torpor versus control. For the relative expression ± SEM values of all 85 miRNA species examined refer to Supplementary Table S2.

The primary goal of this study was to characterize hibernation-specific patterns of miRNA expression in liver and skeletal muscle of the marsupial *D. gliroides*. Of the 85 miRNAs successfully quantified by RT-qPCR, 35 miRNAs were significantly down-regulated in liver during torpor, with miRNA relative abundances decreasing by 0.21–0.72 fold change from the control condition (*p* < 0.05; Supplementary Table S2 and Fig. 1). A different pattern was observed in skeletal muscle, where seven miRNAs showed elevated expression during torpor, increasing by 0.32–1.52 fold change from control levels (dgl-miR-1a-1-5p, dgl-miR-1b-5p, dgl-miR-139-5p, dgl-miR-181a-3p, dgl-miR-190a-5p, dgl-miR-483-5p, and dgl-miR-99b-5p), whereas, 4 miRNAs showed decreased expression of 0.33-0.43 fold change compared to control (dgl-miR-16-1-3p, dgl-miR-185-5p, dgl-miR-22-5p, and dgl-miR-33a-5p) (*p* < 0.05; Supplementary Table S2 and Fig. 1). Of these, three miRNAs showed a pattern of decreased expression that was consistent in both tissues during torpor (dgl-miR-16-3p, dgl-miR-22-5p, and dgl-miR-185-5p). In contrast, four miRNAs (dgl-miR-1a1-5p, dgl-miR-1b-5p, dgl-miR-99b-5p, and dgl-miR-139-5p) showed a torpor-specific increase in skeletal muscle, but with a concomitant decrease in liver tissue.

### Bioinformatic analyses of pathways regulated by miRNA during torpor

**Figure 2:**
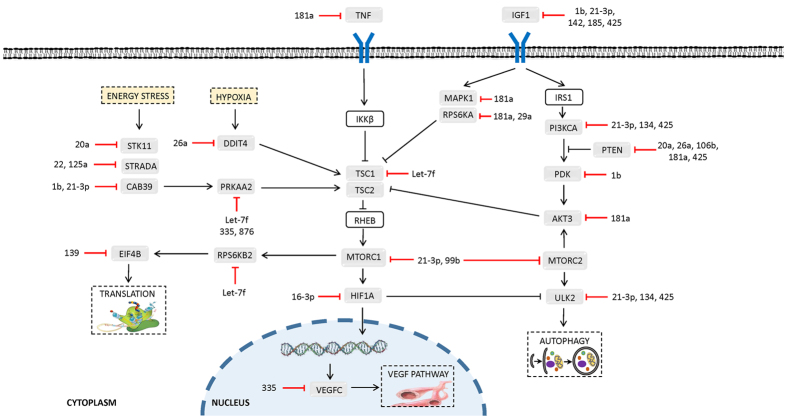
DIANA mirPath analysis of 35 torpor-repressed miRNAs in *D. gliroides* liver predicted that 32 genes involved in mTOR signalling are targeted by 23 miRNAs from this group. This figure illustrates which genes are predicted to be upregulated during torpor (grey boxes) and their putative miRNA regulators (red bars indicate decreased miRNA expression during torpor) within the context of a simplified Akt/mTOR signalling pathway. Genes in white boxes were not predicted targets of the 35 suppressed miRNAs in liver.

**Figure 3:**
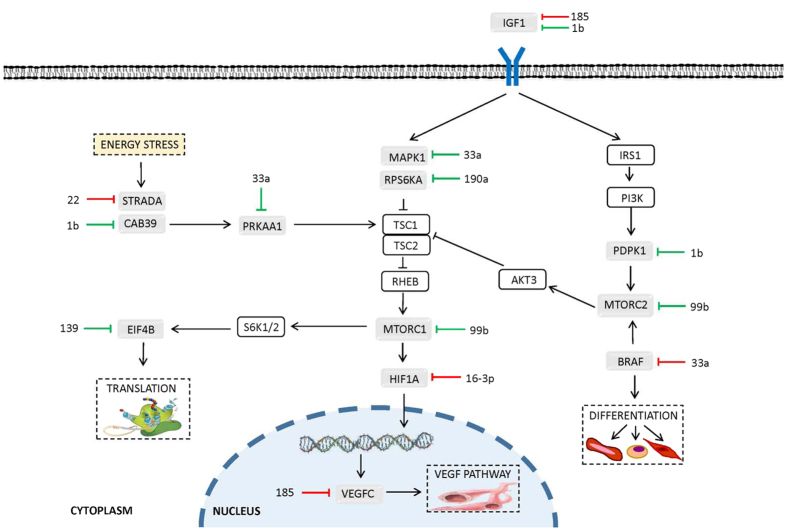
DIANA mirPath analysis of eleven miRNAs showing torpor-specific expression in *D. gliroides* skeletal muscle predicted that twelve genes involved in mTOR signalling are targeted by eight miRNAs from this group. Predicted target genes (grey boxes) and their putative miRNA regulators (green bars indicate increased miRNA expression and red bars indicate decreased miRNA expression during torpor) are shown in the context of a simplified Akt/mTOR signalling pathway. Genes in white boxes were not predicted targets of the eleven differentially expressed miRNAs in skeletal muscle.

MicroRNAs with altered expression during torpor in *D. gliroides* liver and skeletal muscle were subjected to bioinformatic target enrichment analyses using DIANA mirPath v2.0. This program identifies the potential KEGG pathways that are collectively targeted by the query miRNAs. DIANA mirPath indicated that decreased expression of miRNAs in liver of torpid animals were most closely associated with three signalling pathways [1] MAPK signalling (*p* = 8.09E^−25^), with 96 genes thought to be targeted by 32 of the 35 down-regulated miRNAs; [2] mTOR signalling (*p* = 3.09E^−20^), with 32 genes targeted by 23 of 35 torpor-sensitive miRNAs (Fig. 2); and [3] PI3K/Akt signalling (*p* = 7.44E^−07^), with 110 genes targeted by 33 of 35 differentially expressed miRNAs (Table 1, Fig. 2). In skeletal muscle, eleven torpor-associated miRNAs were linked to pathways of [1] focal adhesion (*p* = 7.44E^−07^), with 29 genes targeted by seven miRNAs; [2] ErbB signalling (*p* = 9.60E^−06^), with sixteen genes targeted by seven miRNAs; and [3] mTOR signalling (*p* = 1.64E^−05^), involving twelve genes and eight miRNAs (Table 1 and Fig. 3).

### Conservation of miRNAs across vertebrate taxa

To provide support for the conservation of these miRNAs in *D. gliroides*, a species that is not genome-sequenced, multiple sequence alignments were performed on orthologous pre-miRNA sequences from *M. musculus*, *M. domestica*, and *G. gallus*. In cases where the sequences from these species were not annotated in NCBI GenBank, the alignments were instead performed using sequences from *Homo sapiens*, *Bos taurus*, *Ornithorhynchus anatinus*, *Eptesicus fuscus, Equus caballus, Ovis aries, Sus scofa,* or *Danio rerio.* Alignments were assessed for percent identity of the mature miRNA sequence; only miRNAs with a high degree of conservation in the mature miRNA sequence were quantified in the *D. gliroides* samples. Of the 85 miRNAs analyzed in this study, 50 showed 100% conservation in the mature sequence across taxa, 18 showed at least 90% identity, ten miRNAs had between 80–89% identity, and a small subset of seven mature miRNAs displayed between 70–80% identity across distant species.

## Discussion

The marsupial *D. gliroides* has evolved behavioural, physiological, and biochemical adaptations that facilitate entry into a hypometabolic state during periods of cold exposure and food deprivation^11^. Various studies have examined the ecological, phylogenetic, and physiological nature of *D. gliroides* hypometabolism, but little work has gone into characterizing the biochemical and molecular adaptations of its hibernation response^11^,^42^,^43^,^44^. The phenomenon of hibernation involves the intricate coordination of various biological functions to conserve energy, mainly through the strong depression of cellular ATP-expensive processes such as gene transcription, protein synthesis, transmembrane ion pumping, and others^2^. Recent findings have identified miRNAs as key orchestrators of these cellular adaptations via their actions in inhibiting mRNA translation and/or directing mRNA transcripts into degradation^28^,^45^. The nature of miRNAs makes them excellent master cellular regulators of stress responses: [1] they can exert broad controls over many different genes due to their target promiscuity and dynamic structures^31^, [2] they are easily inducible and readily reversible^27^, and [3] they can preserve mRNA transcripts by sequestering them into RISCs and P-bodies during hypometabolism and then make them available for translation upon arousal^32^. The present study aimed to expand our understanding of miRNA regulation of hibernation by examining the expression responses of 85 highly conserved miRNAs in the liver and skeletal muscle of *D. gliroides*. Although *D. gliroides* is not genome sequenced, miRNA analysis was possible because of the very high degree of evolutionary conservation of miRNAs across species, not only within mammals but across all vertebrates^46^. However, given the paucity of experimentally-validated miRNA-mRNA interactions, pathway analyses in this study necessitated the use of predicted miRNA interactions in mice from the algorithm-based target enrichment database, DIANA miRPath.

The liver is the body’s main metabolic center and the site of many important functions including ketogenesis, gluconeogenesis, processing of nutrients, detoxification reactions, and the synthesis of proteins that are secreted for use elsewhere in the body. *D. gliroides* liver showed significant decreases in the relative abundances of 35 miRNAs during torpor (Fig. 1). This strong miRNA suppression implies that during torpor there is an increased translation of a wide variety of mRNA transcripts that are targets of these miRNAs. It was predicted that the suppressed miRNAs in liver are involved in regulating genes of the MAPK, mTOR, and PI3K-Akt signalling pathways, suggesting that these pathways become activated during marsupial torpor, and are necessary for regulating metabolic reorganization and proper hepatic function in the hypometabolic state (Table 1).

The regulation of MAPK and PI3K-Akt signalling pathways by miRNA could help to induce the metabolic reorganization required during torpor, since the protein kinase cascades of these pathways use reversible protein phosphorylation to induce rapid changes in many cellular functions. MAPK signal cascades relay stimuli, such as stress signals, from extracellular receptors to nuclear effectors in order to initiate appropriate cellular responses as diverse as cell division, growth, apoptosis and metabolism^47^,^48^. Indeed, 96 genes in the MAPK signalling pathway are known targets for 32 of the 35 torpor-sensitive miRNAs identified in *D. gliroides* liver (Table 1). This corresponds with findings from placental hibernators, Richardson’s and thirteen-lined ground squirrels, that also show differential-regulation of MAPK signalling in an organ-specific fashion during torpor^49^,^50^. Collectively, these results indicate that MAPK signalling plays a conserved role in facilitating torpor. Furthermore, roles for the suppression of specific miRNAs can also be suggested. For example, overexpression of miR-34a has been documented to elicit pro-apoptotic responses through the reduced expression of MAP3K9 and other MAPK family members^51^. Therefore, the observed 0.38 ± 0.04 fold change reduction in the relative expression of liver dgl-miR-34a during torpor suggests that it could be contributing to the predicted activation of MAPK signalling and the inhibition of pro-apoptotic mechanisms (Supplementary Table S2 and Fig. 1). Indeed, it has been shown that diverse animals, from hibernating ground squirrels to estivating milk snails, suppress apoptosis and promote cell survival pathways such as MAPK, during prolonged periods of hypometabolism^52^,^53^.

PI3K-Akt signalling was also predicted to be differentially regulated in liver of torpid marsupials, with the data showing that 110 genes in this pathway are targeted by 32 of the miRNAs suppressed during torpor (Table 1). Akt signalling exerts positive controls on transcription, protein synthesis, and cell survival through the phosphorylation and activation of key protein kinases^48^,^54^ (Fig. 2). Of the suppressed miRNAs that target PI3K-Akt signalling, the 0.57 ± 0.03 fold change decrease in dgl-miR-29a in torpid livers is of particular interest (Supplementary Table S2 and Fig. 1). The overexpression of miR-29a has been shown to suppress PI3K regulatory subunits and act as a direct negative regulator of Akt-3 signalling^55^. This suggests that the measured reduction in dgl-miR-29a could be directly involved in coordinating the activation of Akt signalling during torpor. The Akt pathway influences the induction of mTOR signalling and the subsequent initiation of ribosomal biogenesis and protein translation through the activation of eIF4E^56^. In *D. gliroides*, an induction of mTOR by Akt signaling could be complemented by the predicted targeting of 32 mTOR pathway genes through the action of 23 miRNAs that were suppressed in liver (Table 1 and Fig. 2). The predicted activation of Akt signalling during marsupial torpor corresponds with findings from other hypometabolic animal models that also showed Akt activation through the increased protein abundance levels of key Akt elements^53^,^57^. This cross-species trend of Akt signalling emphasizes the cell survival mechanisms that Akt signalling controls and their importance in facilitating torpor.

The trend of activation of selected elements of liver metabolism that is predicted from our miRNA data is of particular interest for hibernating *D. gliroides* since marsupials may lack brown adipose tissue^20^, the crucial thermogenic tissue used by eutherian mammals to rewarm their bodies during arousal from torpor^58^,^59^. Here, we present a potential liver-centered compensatory mechanism for marsupial thermoregulation during torpor. In addition to the classical shivering thermogenesis performed by skeletal muscles, a role for liver metabolism in heat generation has also been proposed for marsupials^24^,^25^. The thermoregulatory mechanisms invoked during arousal seem to represent the main difference in physiology between marsupial hibernators and their eutherian counterparts^5^. A study of the non-hibernating marsupial *M. domestica* found a 48% increase in liver mass and a 20% increase in total liver mitochondrial volume following cold-acclimation, thereby increasing the tissue’s capacity to produce ATP aerobically^24^. The increased liver activity observed in *M. domestica* during cold exposure corresponds with the predicted activation of *D. gliroides* liver metabolism based on the observed torpor-responsive differential suppression of all miRNA species examined in this study. Indeed, during cold exposure, the liver’s oxidative capacity has been found to contribute up to 25% of the total heat generated under basal metabolic conditions^60^. The non-shivering mechanisms of hepatic heat production, such as futile substrate cycling, uncoupling of liver mitochondrial oxidative phosphorylation, and non-specific proton leaks are based on various signalling mechanisms^24^,^61^,^62^. Therefore, the predicted activation of MAPK, PI3K-Akt, and mTOR signalling in *D. gliroides* corresponds with a proposed role for marsupial liver in facilitating heat production.

The hibernation-specific changes in miRNA expression measured in skeletal muscle were less profound than those observed in liver, though this might be expected as the structurally and functionally different liver tissue has been documented as the more metabolically active^63^. Eleven miRNAs were found to be differentially expressed in torpid skeletal muscle and could contribute to differential regulation of various muscle processes such as a flexible reorganization of cellular morphology, fuel metabolism, contractile factors, and organelles to adapt to varying physiological and environmental demands^64^,^65^. Moreover, skeletal muscle plays a critical role in thermoregulation, especially in marsupials that rely on the mechanical heat generated from the rapid muscular contractions during shivering thermogenesis and the metabolic heat generated from muscular non-shivering thermogenesis to arouse from torpor^18^,^66^. Thus, it is essential that skeletal muscle suffers minimal disuse atrophy during torpor. As such, the activation of specific genes may be necessary to maintain the muscle contractile apparatus during torpor, and to ensure that skeletal muscle does not undergo major damage, thus allowing proper skeletal muscle functions to be rapidly restored upon arousal. Previous studies have determined that hibernating animals, including bats and ground squirrels, experience little to no muscle disuse atrophy during extended periods of hypometabolism^67^,^68^. Furthermore, an examination of miRNA expression in torpid little brown bats highlighted differentially expressed miRNAs involved in mediating muscle atrophy resistance^14^.

Of the eleven miRNAs that changed in marsupial muscle during torpor, seven were found to be upregulated and four downregulated (Fig. 1). Bioinformatic target enrichment analyses predicted that the main KEGG pathways targeted by the changing muscle miRNAs were [1] focal adhesion, [2] ErbB, and [3] mTOR pathways (Table 1). Twenty nine genes involved in focal adhesion were targeted by a network of seven muscle-specific miRNAs, a subset of which were upregulated during torpor. Indeed, focal adhesion has been implicated as an important processes for the structural and functional maintenance of skeletal muscle integrity and for transmission of contractile lateral forces^69^,^70^. The regulatory controls elicited by miRNAs may act to coordinate physiological changes that reduce skeletal muscle damage over repeated torpor/arousal cycling. In particular, the overexpression of the muscle-specific miR-1 family has been shown to protect against atrophy through the regulation of skeletal myogenesis, muscle differentiation, and maintenance mechanisms^71^. MiRNAs dgl-miR-1a and dgl-miR-1b were both upregulated, by 0.61 ± 0.08 and 0.65 ± 0.07 fold change respectively, in response to torpor in skeletal muscle (Supplementary Table S2 and Fig. 1). MiR-1 promotes myogenesis through the direct and indirect targeting of various myogenic markers, including MEF2 transcription factors^72^. MEF2s are necessary for preventing muscle atrophy as their inactivation leads to the loss of oxidative capacity and a reduction in slow, type-I myosin heavy chain expression^73^. Moreover, an examination of MEF2s in hibernating thirteen-lined ground squirrels found that both mRNA and protein were upregulated during torpor^68^,^74^. This activation of pro-myogenic activity observed in hibernating squirrels and predicted in hibernating marsupials counteracts the physical inactivity of muscle experienced during winter months and likely helps maintain muscle mass and function. Furthermore, this upregulation appears to be a common trend amongst hibernators since miR-1a was also overexpressed in the skeletal muscle of hibernating little brown bats^14^.

Twelve genes in the mTOR signalling pathway were targeted by eight differentially regulated skeletal muscle miRNAs, half of which were upregulated and half were downregulated (Table 1; Fig. 3). The predicted regulation of mTOR corresponds with other studies that have implicated mTOR signalling as one of the key molecular pathways involved in the development and maintenance of skeletal muscle mass^75^. Moreover, a study of thirteen-lined ground squirrels identified inhibition of mTOR signalling during hibernation in skeletal muscle^76^. Interestingly, mTOR signalling was a miRNA targeted pathway identified in both liver and skeletal muscle responses to marsupial torpor although the two tissues displayed different activation patterns. The miRNA response in liver during torpor suggested an activation of mTOR signalling and genes involved in promoting translation, VEGF-mediated vasculogenesis and angiogenesis, as well as autophagy (Fig. 2). Similarly, eight of the miRNAs that were differentially expressed in skeletal muscle tissue were also likely involved in regulating the translation of mTOR pathway genes, and regulating the VEGF pathway and differentiation (Fig. 3). These results demonstrate the varying tissue-specific effects that miRNAs can have on the same signalling pathway in response to the same stress. Overall, the results from DIANA mirPath are suggestive of a general activation and regulation of the highlighted pathways in liver and skeletal muscle of torpid marsupials and implicates their involvement in facilitating the metabolic changes necessary to survive torpor.

This study is the first to explore the role of miRNA as a regulator of torpor in the hibernating marsupial *D. gliroides*. Of the 85 miRNAs analyzed, 35 showed significantly reduced expression in liver, whereas in skeletal muscle, seven had reduced expression, and four miRNAs were upregulated (*p* < 0.05). Overall, the results show a tissue-specific response by miRNAs during marsupial hibernation and suggest that they may play a pivotal role in initiating and facilitating metabolic rate depression and/or other adjustments needed for sustained survival in a hypometabolic state. Torpor-specific miRNAs in liver suggested that MAPK, PI3K-Akt, and mTOR pathways play an active role in liver, potentially representing a compensatory mechanism for thermoregulation during torpor. Differentially expressed skeletal muscle miRNAs were predicted to target focal adhesion, ErbB, and mTOR signalling pathways that may play a role in maintaining muscle mass and function during the inactive periods of torpor. This study broadens our current understanding of the adaptive molecular mechanisms and miRNA involvement in safely and reversibly transitioning into extreme hypometabolic states.

## Methods

### Treatment of animals

Adult *D. gliroides* individuals were captured near Valdivia, Chile (39°48′S, 73°14′W; 9 m.a.s.l) during the austral summer (January-February) of 2014. Bananas and yeast were used as bait in modified tomahawk traps that were set up 1m above the ground in trees and shrubs. Upon capture, individuals were immediately transported to the laboratory where they were housed in plastic cages of 45 × 30 × 20 cm^3^ with 2 cm of bedding. All individuals were maintained in a climate controlled chamber (PiTec Instruments, Chile) at 20 ± 1 °C and with a 12 h: 12 h light:dark photoperiod for two weeks. Animals were fed a mix of mealworms and fruits with water *ad libitum*. After two weeks of acclimation, two experimental treatments were run in parallel with randomly selected individuals for torpor and control conditions. Active control animals were sampled from the conditions described above. In order to induce torpor, animals were subjected to a gradual decrease of ambient temperature (−1 °C every 12 h) until 10 °C was reached. Torpor incidence was verified with body temperature measurements and visual observation, as described in Franco *et al.* several times daily between 09:00–17:00^77^. Once in torpor, animals were kept under experimental conditions for four days with daily offers of food and water. Individuals in torpor versus active were then euthanized following protocols approved by the Committee on the Ethics of Animal Experiments of the Universidad Austral de Chile. Tissue samples were rapidly excised and immediately frozen in liquid nitrogen; skeletal muscle tissue was mixed leg muscle from the left, hind leg. Frozen samples were packed in a dry shipper and air freighted to Carleton University. All animal capture, handling and maintenance procedures were in accordance with the guidelines of the American Society of Mammalogists^78^ and were approved by the Chilean Agriculture and Livestock Bureau (SAG: Servicio Agrícola y Ganadero de Chile, permit resolution No. 1054/2014).

### Total RNA isolation

Isolation of RNA was conducted as previously described^37^. For each sample, ~50 mg of tissue (liver or skeletal muscle) was briefly homogenized in 1 mL Trizol (Invitrogen) using a Polytron PT1200 homogenizer; a 200 μL aliquot of chloroform was then added, and samples were centrifuged at 10,000 rpm for 15 min at 4 °C. The upper aqueous phase was transferred to a new centrifuge tube and 500 μL of isopropanol was added, mixing well. RNA was allowed to precipitate for 10 min on ice, then samples were centrifuged at 12,000 rpm for 15 min at room temperature. Pellets were washed with 1 mL of 70% ethanol, then centrifuged at 7,500 rpm for 5 min. Ethanol was decanted and RNA precipitates were allowed to air-dry for 10 min, followed by resuspending RNA in 50 μL of RNase-free water. Quality of RNA isolations was verified by measuring the 260/280 nm ratio with a NanoDrop spectrometer (Fisher Scientific, Wilmington, DE); only samples with 260/280 nm >1.8 were analyzed. Total RNA integrity was assessed by the presence of sharp bands for 28S and 18S ribosomal RNA on a 1% agarose gel stained with SYBR Green. All RNA samples were standardized to a final concentration of 1 μg/μL with RNase-free water. Samples were frozen at −20 °C until use.

### MiRNA polyadenylation and stem-loop reverse transcription

RNA samples were prepared for miRNA analysis as previously described^35^. Polyadenylation of microRNA was carried out using PolyA tailing kit from Epi-Bio (Cat# PAP5104H; Epicentre, Madison, WI, USA). Each 10 μL reaction contained 3 μg total RNA, 1 mM ATP, and 0.5 μL (2 U) of *E. coli* poly (A) polymerase in buffered solution (0.1 M Tris-HCl pH 8.0, 0.25 M NaCl, and 10 mM MgCl_2_). Reactions were incubated at 37 °C for 30 min to adenylate, 95 °C for 5 min to arrest, and then chilled on ice. For reverse transcription, the polyadenylated products (10 μL samples) were combined with 5 μL of 250 pM stem-loop adapter primers (Supplementary Table S1) then heated to 95 °C for 5 min to denature RNA, cooled to 60 °C for 5 min to allow annealing, and placed on ice for 1 min. Mastermix was added such that each 25 μL sample contained 2 units of mouse Maloney leukemia virus (M-MLV) reverse transcriptase, 1 mM of each deoxynucleotide triphosphate, 1 mM dithiothreitol (DTT), 50 mM Tris-HCl pH 8.3, 40 mM KCl, and 6 mM MgCl_2_. Samples were incubated at 16 °C for 30 min, 42 °C for 30 min, and 85 °C for 5 min. Products were serially diluted and frozen at −20 °C.

### MiRNA sequence conservation

The miRNA-specific forward primers were designed based on annotated miRNA sequences from the *Mus musculus* genome and the precursor miRNA stem-loop sequences of *M. musculus* were obtained from miRBase (Release 21)^79^. To validate the use of these primers for RT-qPCR of miRNAs from *D. gliroides*, the precursor miRNA stem-loop sequences of *M. musculus* were subjected to NCBI BLASTn to assess the miRNA conservation across taxa. Multiple sequence alignments were performed with EMBL-EBI Clustal W with the stem-loop pre-miRNA sequences from *M. musculus*, *M. domestica*, and *G. gallus*. In cases where these sequences were unavailable or unannotated in NCBI GenBank, the alignments were conducted using sequences from other diverse vertebrates, including *Homo sapiens*, *Bos taurus*, *Ornithorhynchus anatinus*, *Takifugu rubripes,* and *Danio rerio.*

### Relative miRNA quantification

All RT-qPCR assays were performed as previously described^80^ using a BioRad MyIQ2 Detection System (BioRad, Hercules, CA, USA), following MIQE guidelines^81^. MiRNA-specific forward primers were designed using the previously described method to amplify conserved miRNAs^82^. These were used in conjunction with a universal reverse primer designed to amplify all products of reverse-transcription (Supplementary Table S1). Primers were synthesized by Integrated DNA Technologies (Coralville, Iowa, USA). To ensure the amplification of a single PCR product only, all PCR assays were subjected to post-run melt-curve analysis; reactions that amplified multiple non-specific products were rejected.

### Relative Quantification and Statistics

The comparative ΔΔCq method was used to calculate relative levels of miRNA expression^81^. Raw Cq values were transformed to the 2^-Cq^ form, such that the miRNA of interest could be normalized to the reference gene, *U6 snRNA. U6 snRNA* was experimentally determined to be a suitable reference gene for this study based on its stable expression in *D. gliroides* liver and skeletal muscle under both control and torpor conditions and using the calculations previously described by Schmittgen and Livak^83^. Data was collected and analyzed as mean relative expression (mean ± SEM, where *n* = 4 independent biological replicates of tissue from different animals with two highly comparable technical replicates for each quantification). Differences in miRNA relative expression between control and torpor conditions were considered significant when a Student’s *t*-test resulted in *p* < 0.05.

### Predictions of miRNA-targeted pathways

MiRNAs that were differentially expressed in the torpid state were analyzed using the DNA Intelligent Analysis (DIANA) miRPath v2.0 program with *M. musculus* miRNA homologs (microT-CDS setting with threshold of 0.8)^84^,^85^. This bioinformatics tool uses *in silico* predicted miRNA-mRNA interactions and Kyoto Encyclopedia of Genes and Genomes (KEGG) to identify cellular pathways enriched with target genes. Two groups were queried separately: [1] the 35 miRNAs with lower expression in liver from torpid animals, and [2] the 11 differentially regulated skeletal muscle miRNAs.

## Additional Information

**How to cite this article**: Hadj-Moussa, H. *et al.* The hibernating South American marsupial, *Dromiciops gliroides*, displays torpor-sensitive microRNA expression patterns. *Sci. Rep.* **6**, 24627; doi: 10.1038/srep24627 (2016).

## Supplementary Material

Supplementary Information

## Figures and Tables

**Table 1:** Results from DIANA mirPath v2.0 prediction of KEGG pathways influenced by the torpor-specific expression of miRNAs in *D. gliroides* liver and skeletal muscle tissues.

Tissue	KEGG Pathway	MicroRNAs (involved/queried)	# of Genes Targeted	*p*-value
Liver	MAPK signalling	32/35	96	8.09E^−25^
	mTOR signalling	23/35	32	3.09E^−20^
	PI3k-Akt signalling	32/35	111	6.88E^−15^
Skeletal Muscle	Focal adhesion	7/11	29	7.44E^−07^
	ErbB signalling	7/11	16	9.60E^−06^
	mTOR signalling	8/11	12	1.64E^−05^

The analysis was performed using MicroT-CDS *in silico* prediction with threshold of 0.8 and results were merged by genes union.

## References

[b1] Storey K. B. & Storey J. M. Aestivation: signaling and hypometabolism. J. Exp. Biol. 215, 1425–33 (2012). 22496277 10.1242/jeb.054403

[b2] Storey K. B. Regulation of hypometabolism: insights into epigenetic controls. J. Exp. Biol. 218, 150–159 (2015). 25568462 10.1242/jeb.106369

[b3] McNab B. K. The comparative energetics of neotropical marsupials. J. Comp. Physiol. B 125, 115–128 (1978).

[b4] Ruf T. & Geiser F. Daily torpor and hibernation in birds and mammals. Biol. Rev. Camb. Philos. Soc. 90, 891–926 (2014). 10.1111/brv.12137 PMC4351926 25123049

[b5] Cortés P. A., Franco M., Moreno-Gómez F. N., Barrientos K. & Nespolo R. F. Thermoregulatory capacities and torpor in the South American marsupial, Dromiciops gliroides. J. Therm. Biol. 45, 1–8 (2014). 25436944 10.1016/j.jtherbio.2014.07.003

[b6] Geiser F. Metabolic rate and body temperature reduction during hibernation and daily torpor. Annu. Rev. Physiol. 66, 239–274 (2004). 14977403 10.1146/annurev.physiol.66.032102.115105

[b7] Storey K. B. Out cold: Biochemical regulation of mammalian hibernation - A mini-review. Gerontology 56, 220–230 (2010). 19602865 10.1159/000228829

[b8] Bozinovic F., Ruiz G. & Rosenmann M. Energetics and torpor of a South American ‘living fossil’, the microbiotheriid Dromiciops gliroides. J. Comp. Physiol. B. 174, 293–7 (2004). 14760502 10.1007/s00360-004-0414-8

[b9] Mitchell K. J. *et al.* Molecular phylogeny, biogeography, and habitat preference evolution of marsupials. Mol. Biol. Evol. 31, 2322–30 (2014). 24881050 10.1093/molbev/msu176

[b10] Marshall L. G. Dromiciops australis. Am. Soc. Mammal. 99, 1–5 (1978).

[b11] Nespolo R. F., Verdugo C., Cortés P. A. & Bacigalupe L. D. Bioenergetics of torpor in the Microbiotherid marsupial, Monito del Monte (*Dromiciops gliroides*): The role of temperature and food availability. J. Comp. Physiol. B Biochem. Syst. Environ. Physiol. 180, 767–773 (2010). 10.1007/s00360-010-0449-y 20165853

[b12] Storey K. B. & Storey J. M. Metabolic rate depression: the biochemistry of mammalian hibernation. Adv. Clin. Chem. 52, 77–108 (2010). 21275340

[b13] Güller I. & Russell A. P. MicroRNAs in skeletal muscle: their role and regulation in development, disease and function. J. Physiol. 588, 4075–4087 (2010). 20724363 10.1113/jphysiol.2010.194175 PMC3002442

[b14] Kornfeld S. F., Biggar K. K. & Storey K. B. Differential expression of mature microRNAs involved in muscle maintenance of hibernating little brown bats, *Myotis lucifugus*: a model of muscle atrophy resistance. Genomics. Proteomics Bioinformatics 10, 295–301 (2012). 23200139 10.1016/j.gpb.2012.09.001 PMC5054200

[b15] Nicol S. C. Non-shivering thermogenesis in the potoroo, *Potorous tridactylus* (Kerr). Comp. Biochem. Physiol. Part C, Comp. 59, 33–37 (1978). 10.1016/0306-4492(78)90008-4 24519

[b16] Ikonomopoulou M. P. & Rose R. W. The Development of Endothermy during Pouch Life in the Eastern Barred Bandicoot (Perameles gunnii), a Marsupial. Physiol. Biochem. Zool. Ecol. Evol. Approaches 79, 468–473 (2006). 10.1086/502819 16691513

[b17] Loudon A., Rothwell N. & Stock M. Brown fat, thermogenesis and physiological birth in a marsupial. Comp. Biochem. Physiol. – Part A Physiol. 81, 815–819 (1985). 10.1016/0300-9629(85)90912-0 2863071

[b18] Rose R. W., West A. K., Ye J., McCormack G. H. & Colquhoun E. Q. Nonshivering thermogenesis in a marsupial (the Tasmanian Bettong Bettongia gaimardi) is not attributable to brown adipose tissue. Physiol. Biochem. Zool. Ecol. Evol. Approaches 72, 699–704 (1999). 10.1086/316709 10603333

[b19] Nicol S. C., Pavlides D. & Andersen N. A. Nonshivering thermogenesis in marsupials: Absence of thermogenic response to β3-adrenergic agonists. Comp. Biochem. Physiol. - A Physiol. 117, 399–405 (1997). 9172391 10.1016/s0300-9629(96)00357-x

[b20] Hayward J. S. & Lisson P. A. Evolution of brown fat: its absence in marsupials and monotremes. Can. J. Zool. 70, 171–179 (1992).

[b21] Jastroch M., Withers K. & Klingenspor M. Uncoupling protein 2 and 3 in marsupials: identification, phylogeny, and gene expression in response to cold and fasting in Antechinus flavipes. Physiol. Genomics 17, 130–139 (2004). 14970361 10.1152/physiolgenomics.00165.2003

[b22] Jastroch M. *et al.* Marsupial uncoupling protein 1 sheds light on the evolution of mammalian nonshivering thermogenesis. Physiol. Genomics 32, 161–9 (2008). 17971503 10.1152/physiolgenomics.00183.2007

[b23] Polymeropoulos E. T., Jastroch M. & Frappell P. B. Absence of adaptive nonshivering thermogenesis in a marsupial, the fat-tailed dunnart (*Sminthopsis crassicaudata*). J. Comp. Physiol. B Biochem. Syst. Environ. Physiol. 182, 393–401 (2012). 10.1007/s00360-011-0623-x 22002052

[b24] Villarin J. J., Schaeffer P. J., Markle R. A. & Lindstedt S. L. Chronic cold exposure increases liver oxidative capacity in the marsupial Monodelphis domestica. Comp. Biochem. Physiol. Part A Mol. Integr. Physiol. 136, 621–630 (2003). 10.1016/s1095-6433(03)00210-1 14613790

[b25] Stoner H. B. The role of the liver in non-shivering thermogenesis in the rat. J. Physiol. 232, 285–296 (1973). 4727083 10.1113/jphysiol.1973.sp010270 PMC1350455

[b26] Stone G. N. & Purvis A. Warm-up rates during arousal from torpor in heterothermic mammals: physiological correlates and a comparison with heterothermic insects. J. Comp. Physiol. B 162, 284–295 (1992). 1613167 10.1007/BF00357536

[b27] Biggar K. K. & Storey K. B. The emerging roles of microRNAs in the molecular responses of metabolic rate depression. J. Mol. Cell Biol. 3, 167–175 (2011). 21177365 10.1093/jmcb/mjq045

[b28] Leung A. K. L. & Sharp P. A. MicroRNA functions in stress responses. Mol. Cell 40, 205–215 (2010). 20965416 10.1016/j.molcel.2010.09.027 PMC2996264

[b29] Bartel D. P. MicroRNAs: target recognition and regulatory functions. Cell 136, 215–33 (2009). 19167326 10.1016/j.cell.2009.01.002 PMC3794896

[b30] Ebert M. S. & Sharp P. A. Roles for microRNAs in conferring robustness to biological processes. Cell 149, 515–24 (2012). 22541426 10.1016/j.cell.2012.04.005 PMC3351105

[b31] Bartel D. P. MicroRNAs genomics, biogenesis, mechanism, and function. Cell 116, 281–297 (2004). 14744438 10.1016/s0092-8674(04)00045-5

[b32] Huang Y. *et al.* Biological functions of microRNAs: a review. J. Physiol. Biochem. 67, 129–39 (2011). 20981514 10.1007/s13105-010-0050-6

[b33] Biggar K. K., Kornfeld S. F., Maistrovski Y. & Storey K. B. MicroRNA regulation in extreme environments: differential expression of microRNAs in the intertidal snail *Littorina littorea* during extended periods of freezing and anoxia. Genomics. Proteomics Bioinformatics 10, 302–9 (2012). 23200140 10.1016/j.gpb.2012.09.002 PMC5054212

[b34] Biggar K. K. & Storey K. B. Evidence for cell cycle suppression and microRNA regulation of cyclin D1 during anoxia exposure in turtles. Cell Cycle 11, 1705–13 (2012). 22510561 10.4161/cc.19790

[b35] Biggar K. K. & Storey K. B. Identification and expression of microRNA in the brain of hibernating bats, Myotis lucifugus. Gene 544, 67–74 (2014). 24768722 10.1016/j.gene.2014.04.048

[b36] Chen M., Zhang X., Liu J. & Storey K. B. High-throughput sequencing reveals differential expression of miRNAs in intestine from sea cucumber during aestivation. Plos One 8, e76120 (2013). 24143179 10.1371/journal.pone.0076120 PMC3797095

[b37] Luu B. E. & Storey K. B. Dehydration triggers differential microRNA expression in *Xenopus laevis* brain. Gene 573, 64–69 (2015). 26169019 10.1016/j.gene.2015.07.027

[b38] Morin P., Dubuc A. & Storey K. B. Differential expression of microRNA species in organs of hibernating ground squirrels: a role in translational suppression during torpor. Biochim. Biophys. Acta 1779, 628–33 (2008). 18723136 10.1016/j.bbagrm.2008.07.011

[b39] Wu C. W., Biggar K. K. & Storey K. B. Dehydration mediated microRNA response in the African clawed frog *Xenopus laevis*. Gene 529, 269–275 (2013). 23958654 10.1016/j.gene.2013.07.064

[b40] Wu C. W., Biggar K. K. & Storey K. B. Expression profiling and structural characterization of microRNAs in adipose tissues of hibernating ground squirrels. Genomics. Proteomics Bioinformatics 12, 284–291 (2014). 25526980 10.1016/j.gpb.2014.08.003 PMC4411486

[b41] Yuan L. *et al.* Down but not out: The role of microRNAs in hibernating bats. Plos One 10, e0135064 (2015). 26244645 10.1371/journal.pone.0135064 PMC4526555

[b42] Fontúrbel F. E., Silva-Rodríguez E. A., Cárdenas N. H. & Jiménez J. E. Spatial ecology of monito del monte (*Dromiciops gliroides*) in a fragmented landscape of southern Chile. Mamm. Biol. 75, 1–9 (2010).

[b43] Palma R. E. & Spotorno A. E. Molecular systematics of marsupials based on the rRNA 12S mitochondrial gene: the phylogeny of didelphimorphia and of the living fossil microbiotheriid *Dromiciops gliroides* Thomas. Mol. Phylogenet. Evol. 13, 525–35 (1999). 10620411 10.1006/mpev.1999.0678

[b44] Rodríguez-Cabal M. A., Amico G. C., Novaro A. J. & Aizen M. A. Population characteristics of *Dromiciops gliroides* (Philippi, 1893), an endemic marsupial of the temperate forest of Patagonia. Mamm. Biol. 73, 74–76 (2008).

[b45] Biggar K. K. & Storey K. B. Insight into post-transcriptional gene regulation: stress-responsive microRNAs and their role in the environmental stress survival of tolerant animals. J. Exp. Biol. 218, 1281–1289 (2015). 25954040 10.1242/jeb.104828

[b46] Friedman R. C., Farh K. K.-H., Burge C. B. & Bartel D. P. Most mammalian mRNAs are conserved targets of microRNAs. Genome Res. 19, 92–105 (2009). 18955434 10.1101/gr.082701.108 PMC2612969

[b47] Cowan K. J. Mitogen-activated protein kinases: new signaling pathways functioning in cellular responses to environmental stress. J. Exp. Biol. 206, 1107–1115 (2003). 12604570 10.1242/jeb.00220

[b48] Seger R. & Krebs E. G. The MAPK signaling cascade. FASEB J. 9, 726–35 (1995). 7601337

[b49] Rouble A. N., Tessier S. N. & Storey K. B. Characterization of adipocyte stress response pathways during hibernation in thirteen-lined ground squirrels. Mol. Cell. Biochem. 393, 271–282 (2014). 24777704 10.1007/s11010-014-2070-y

[b50] MacDonald J. A. & Storey K. B. Mitogen-activated protein kinases and selected downstream targets display organ-specific responses in the hibernating ground squirrel. Int. J. Biochem. Cell Biol. 37, 679–91 (2005). 15618024 10.1016/j.biocel.2004.05.023

[b51] Tivnan A. *et al.* MicroRNA-34a is a potent tumor suppressor molecule *in vivo* in neuroblastoma. BMC Cancer 11, 33 (2011). 21266077 10.1186/1471-2407-11-33 PMC3038978

[b52] Rouble A. N., Hefler J., Mamady H., Storey K. B. & Tessier S. N. Anti-apoptotic signaling as a cytoprotective mechanism in mammalian hibernation. PeerJ 1, e29 (2013). 23638364 10.7717/peerj.29 PMC3628845

[b53] Ramnanan C. J., Groom A. G. & Storey K. B. Akt and its downstream targets play key roles in mediating dormancy in land snails. Comp. Biochem. Physiol. B. Biochem. Mol. Biol. 148, 245–55 (2007). 17611133 10.1016/j.cbpb.2007.06.002

[b54] Manning B. D. & Cantley L. C. AKT/PKB signaling: navigating downstream. Cell 129, 1261–74 (2007). 17604717 10.1016/j.cell.2007.06.009 PMC2756685

[b55] Wei W. *et al.* miR-29 targets Akt3 to reduce proliferation and facilitate differentiation of myoblasts in skeletal muscle development. Cell Death Dis. 4, e668 (2013). 23764849 10.1038/cddis.2013.184 PMC3698551

[b56] Zhou L. *et al.* 4E-binding protein phosphorylation and eukaryotic initiation factor-4E release are required for airway smooth muscle hypertrophy. Am. J. Respir. Cell Mol. Biol. 33, 195–202 (2005). 15901615 10.1165/rcmb.2004-0411OC PMC1578595

[b57] McMullen D. C. & Hallenbeck J. M. Regulation of Akt during torpor in the hibernating ground squirrel, Ictidomys tridecemlineatus. J. Comp. Physiol. B 180, 927–934 (2010). 20352231 10.1007/s00360-010-0468-8 PMC2957659

[b58] Dawson T. J. & Olson J. M. Thermogenic capabilities of the opossum *Monodelphis domestica* when warm and cold acclimated: Similarities between american and australian marsupials. Comp. Biochem. Physiol. Part A Physiol. 89, 85–91 (1988). 10.1016/0300-9629(88)91143-7 2450718

[b59] Foster D. O. & Frydman M. L. Nonshivering thermogenesis in the rat. II. Measurements of blood flow with microspheres point to brown adipose tissue as the dominant site of the calorigenesis induced by noradrenaline. Can. J. Physiol. Pharmacol. 56, 110–122 (1978). 638848 10.1139/y78-015

[b60] Janský L. Non-shivering thermogenesis and its thermoregulatory significance. Biol. Rev. Camb. Philos. Soc. 48, 85–132 (1973). 4578360 10.1111/j.1469-185x.1973.tb01115.x

[b61] Horwitz B. A. The effect of cold exposure on liver mitochondrial and peroxisomal distribution in the rat, hamster and bat. Comp. Biochem. Physiol. Part A Physiol. 54, 45–48 (1976). 10.1016/s0300-9629(76)80070-9 3343

[b62] Rolfe D. F., Newman J. M., Buckingham J. A., Clark M. G. & Brand M. D. Contribution of mitochondrial proton leak to respiration rate in working skeletal muscle and liver and to SMR. Am. J. Physiol. 276, C692–C699 (1999). 10069997 10.1152/ajpcell.1999.276.3.C692

[b63] Berg J. M., Tymoczko J. L. & Stryer L. *Biochemistry. Each organ has a unique metabolic profile* (2002).

[b64] Fluck M. Functional, structural and molecular plasticity of mammalian skeletal muscle in response to exercise stimuli. J. Exp. Biol. 209, 2239–48 (2006). 16731801 10.1242/jeb.02149

[b65] Hoppeler H. & Desplanches D. Muscle structural modifications in hypoxia. Int. J. Sports Med. 13, S166–S168 (2008). 1483763 10.1055/s-2007-1024628

[b66] Opazo J. C., Nespolo R. F. & Bozinovic F. Arousal from torpor in the chilean mouse-opposum (*Thylamys elegans*): does non-shivering thermogenesis play a role? Comp. Biochem. Physiol. Part A Mol. Integr. Physiol. 123, 393–397 (1999). 10.1016/s1095-6433(99)00081-1 10581704

[b67] Lee K. *et al.* Overcoming muscle atrophy in a hibernating mammal despite prolonged disuse in dormancy: proteomic and molecular assessment. J. Cell. Biochem. 104, 642–56 (2008). 18181155 10.1002/jcb.21653

[b68] Tessier S. N. & Storey K. B. Expression of myocyte enhancer factor-2 and downstream genes in ground squirrel skeletal muscle during hibernation. Mol. Cell. Biochem. 344, 151–62 (2010). 20617369 10.1007/s11010-010-0538-y

[b69] Crossland H. *et al.* Focal adhesion kinase is required for IGF-I-mediated growth of skeletal muscle cells via a TSC2/mTOR/S6K1-associated pathway. Am. J. Physiol. Endocrinol. Metab. 305, E183–93 (2013). 23695213 10.1152/ajpendo.00541.2012 PMC3725543

[b70] Fluck M., Ziemiecki A., Billeter R. & Muntener M. Fibre-type specific concentration of focal adhesion kinase at the sarcolemma: influence of fibre innervation and regeneration. J. Exp. Biol. 205, 2337–2348 (2002). 12124360 10.1242/jeb.205.16.2337

[b71] Safdar A., Abadi A., Akhtar M., Hettinga B. P. & Tarnopolsky M. A. miRNA in the regulation of skeletal muscle adaptation to acute endurance exercise in C57Bl/6J male mice. Plos One 4, e5610 (2009). 19440340 10.1371/journal.pone.0005610 PMC2680038

[b72] Chen J.-F. *et al.* The role of microRNA-1 and microRNA-133 in skeletal muscle proliferation and differentiation. Nat. Genet. 38, 228–233 (2006). 16380711 10.1038/ng1725 PMC2538576

[b73] Bassel-Duby R. & Olson E. N. Signaling pathways in skeletal muscle remodeling. Annu. Rev. Biochem. 75, 19–37 (2006). 16756483 10.1146/annurev.biochem.75.103004.142622

[b74] Tessier S. N. & Storey K. B. Myocyte enhancer factor-2 and cardiac muscle gene expression during hibernation in thirteen-lined ground squirrels. Gene 501, 8–16 (2012). 22513076 10.1016/j.gene.2012.04.004

[b75] Bodine S. C. *et al.* Akt/mTOR pathway is a crucial regulator of skeletal muscle hypertrophy and can prevent muscle atrophy *in vivo*. Nat. Cell Biol. 3, 1014–9 (2001). 11715023 10.1038/ncb1101-1014

[b76] Wu C. W. & Storey K. B. Regulation of the mTOR signaling network in hibernating thirteen-lined ground squirrels. J. Exp. Biol. 215, 1720–1727 (2012). 22539739 10.1242/jeb.066225

[b77] Franco M., Contreras C. & Nespolo R. F. Profound changes in blood parameters during torpor in a South American marsupial. Comp. Biochem. Physiol. - A Mol. Integr. Physiol. 166, 338–342 (2013). 23850720 10.1016/j.cbpa.2013.07.010

[b78] Gannon W. L. & Sikes R. S. Guidelines of the American Society of Mammalogists for the use of wild mammals in research. J. Mammal. 88, 809–823 (2007). 10.1093/jmammal/gyw078 PMC5909806 29692469

[b79] Kozomara A. & Griffiths-Jones S. miRBase: annotating high confidence microRNAs using deep sequencing data. Nucleic Acids Res. 42, D68–D73 (2014). 24275495 10.1093/nar/gkt1181 PMC3965103

[b80] Pellissier F., Glogowski C. M., Heinemann S. F., Ballivet M. & Ossipow V. Lab assembly of a low-cost, robust SYBR green buffer system for quantitative real-time polymerase chain reaction. Anal. Biochem. 350, 310–2 (2006). 16434019 10.1016/j.ab.2005.12.002

[b81] Bustin S. A. *et al.* The MIQE guidelines: minimum information for publication of quantitative real-time PCR experiments. Clin. Chem. 55, 611–22 (2009). 19246619 10.1373/clinchem.2008.112797

[b82] Biggar K. K., Wu C. W. & Storey K. B. High-throughput amplification of mature microRNAs in uncharacterized animal models using polyadenylated RNA and stem-loop reverse transcription polymerase chain reaction. Anal. Biochem. 462, 32–34 (2014). 24929089 10.1016/j.ab.2014.05.032

[b83] Schmittgen T. D. & Livak K. J. Analyzing real-time PCR data by the comparative C(T) method. Nat. Protoc. 3, 1101–1108 (2008). 18546601 10.1038/nprot.2008.73

[b84] Vlachos I. S. *et al.* DIANA miRPath v.2.0: investigating the combinatorial effect of microRNAs in pathways. Nucleic Acids Res. 40, W498–504 (2012). 22649059 10.1093/nar/gks494 PMC3394305

[b85] Reczko M., Maragkakis M., Alexiou P., Grosse I. & Hatzigeorgiou A. G. Functional microRNA targets in protein coding sequences. Bioinformatics 28, 771–776 (2012). 22285563 10.1093/bioinformatics/bts043

