# Supplementary material for: The hibernating South American marsupial, Dromiciops gliroides, displays torpor-sensitive microRNA expression patterns
Source: Sci Rep. 2016 Apr 19;6:24627. doi: 10.1038/srep24627 (PMC4835794; doi:10.1038/srep24627)
Supplement: Supplementary Information [file srep24627-s1.doc]

# The hibernating South American marsupial, *Dromiciops gliroide­­s,* displays torpor-sensitive microRNA expression patterns

Hanane Hadj-Moussa^1,+^, Jason A. Moggridge^1,+^, Bryan E. Luu^1^, Julian F. Quintero-Galvis^2^, Juan Diego Gaitán-Espitia^3^, Roberto F. Nespolo^2^, and Kenneth B. Storey^1,*^

^1^ Department of Biology and Institute of Biochemistry, Carleton University, 1125 Colonel By Drive, Ottawa, Ontario K1S 5B6, Canada

^2^ Instituto de Ciencias Ambientales y Evolutivas, Facultad de Ciencias, Universidad Austral de Chile, Campus Isla Teja, Valdivia, Chile

^3^ CSIRO Oceans & Atmosphere, GPO Box 1538, Hobart 7001, TAS, Australia

^*^ Corresponding author: kenneth.storey@carleton.ca

^+^Indicates equal input into this study

# SUPPLEMENTARY INFORMATION:

**Supplementary Table S1.** Primers used for analysis of miRNA expression in the liver and skeletal muscle of *D. gliroides*, including miRNA-specific forward primers, universal reverse primer, and the stem-loop adapter for reverse-transcription.

| **Target** | **Primer Sequence (5'-3')** |
| --- | --- |
| dgl-miR-let-7f-5p | ACA CTC CAG CTG GGT GAG GTA GTA GAT TG |
| dgl-miR-1a-5p | ACA CTC CAG CTG GGA CAT ACT TCT TTA TA |
| dgl-miR-1b-5p | ACA CTC CAG CTG GGT ACA TAC TTC TTT AC |
| dgl-miR-7a-5p | ACA CTC CAG CTG GGC AAC AAA TCA CAG TC |
| dgl-miR-9-3-5p | ACA CTC CAG CTG GGT CTT TGG TTA TCT AG |
| dgl-miR-10b-5p | ACA CTC CAG CTG GGT ACC CTG TAG AAC CG |
| dgl-miR-15a-5p | ACA CTC CAG CTG GGT AGC AGC ACA TAA TG |
| dgl-miR-15b-5p | ACA CTC CAG CTG GGT AGC AGC ACA TCA TG |
| dgl-miR-16-3p | ACA CTC CAG CTG GGC CAG TAT TAA CTG TG |
| dgl-miR-17-5p | ACA CTC CAG CTG GGC AAA GTG CTT ACA GT |
| dgl-miR-18a-3p | ACA CTC CAG CTG GGT AAG GTG CAT CTA GT |
| dgl-miR-20a-5p | ACA CTC CAG CTG GGT AAA GTG CTT ATA GT |
| dgl-miR-21a-3p | ACA CTC CAG CTG GGT AGC TTA TCA GAC TG |
| dgl-miR-22-5p | ACA CTC CAG CTG GGA GTT CTT CAG TGG CA |
| dgl-miR-23a-5p | ACA CTC CAG CTG GGG GGG TTC CTG GGG AT |
| dgl-miR-26a-5p | ACA CTC CAG CTG GGT TCA AGT AAT CCA GG |
| dgl-miR-27a-5p | ACA CTC CAG CTG GGA GGG CTT AGC TGC TT |
| dgl-miR-28-5p | ACA CTC CAG CTG GGA AGG AGC TCA CAG TC |
| dgl-miR-29a-5p | ACA CTC CAG CTG GGA CTG ATT TCT TTT GG |
| dgl-miR-30a-5p | ACA CTC CAG CTG GGT GTA AAC ATC CTC GA |
| dgl-miR-30c-5p | ACA CTC CAG CTG GGT GTA AAC ATC CTA CA |
| dgl-miR-30d-5p | ACA CTC CAG CTG GGT GTA AAC ATC CCC GA |
| dgl-miR-30e-5p | ACA CTC CAG CTG GGT GTA AAC ATC CTT GA |
| dgl-miR-32-5p | ACA CTC CAG CTG GGT ATT GCA CAT TAC TA |
| dgl-miR-33a-5p | ACA CTC CAG CTG GGG TGC ATT GTA GTT GC |
| dgl-miR-34a-5p | ACA CTC CAG CTG GGT GGC AGT GTC TTA GC |
| dgl-miR-34c-5p | ACA CTC CAG CTG GGA GGC AGT GTA GTT AG |
| dgl-miR-96-5p | ACA CTC CAG CTG GGT TTG GCA CTA GCA CA |
| dgl-miR-99b-5p | ACA CTC CAG CTG GGC ACC CGT AGA ACC GA |
| dgl-miR-106b-5p | ACA CTC CAG CTG GGT AAA GTG CTG ACA GT |
| dgl-miR-124-3-5p | ACA CTC CAG CTG GGC GTG TTC ACA GCG GA |
| dgl-miR-125a-5p | ACA CTC CAG CTG GGT CCC TGA GAC CCT TT |
| dgl-miR-127-5p | ACA CTC CAG CTG GGC TGA AGC TCA GAG GG |
| dgl-miR-129-1-5p | ACA CTC CAG CTG GGA AGC CCT TAC CCC AA |
| dgl-miR-130b-5p | ACA CTC CAG CTG GGA CTC TTT CCC TGT TG |
| dgl-miR-132-5p | ACA CTC CAG CTG GGA ACC GTG GCT TTC GA |
| dgl-miR-133a-5p | ACA CTC CAG CTG GGG CTG GTA AAA TGG AA |
| dgl-miR-133b-5p | ACA CTC CAG CTG GGG CTG GTC AAA CGG AA |
| dgl-miR-134-5p | ACA CTC CAG CTG GGT GTG ACT GGT TGA CC |
| dgl-miR-137-5p | ACA CTC CAG CTG GGA CGG GTA TTC TTG GG |
| dgl-miR-139-5p | ACA CTC CAG CTG GGT CTA CAG TGC ACG TG |
| dgl-miR-142b-5p | ACA CTC CAG CTG GGT CCA TAA AGT AGG AA |
| dgl-miR-144-5p | ACA CTC CAG CTG GGG GAT ATC ATC GTA TA |
| dgl-miR-145a-5p | ACA CTC CAG CTG GGG TCC AGT TTT CCC AG |
| dgl-miR-147-5p | ACA CTC CAG CTG GGT GGA AAC ATT TCT GC |
| dgl-miR-152-5p | ACA CTC CAG CTG GGT AGG TTC TGT GAT AC |
| dgl-miR-181a-5p | ACA CTC CAG CTG GGA ACA TTC AAC GCT GT |
| dgl-miR-181a-3p | ACA CTC CAG CTG GGA CCA TCG ACC GTT GA |
| dgl-miR-185-5p | ACA CTC CAG CTG GGT GGA GAG AAA GGC AG |
| dgl-miR-186-5p | ACA CTC CAG CTG GGC AAA GAA TTC TCT TT |
| dgl-miR-190a-5p | ACA CTC CAG CTG GGT GAT ATG TTT GAT AT |
| dgl-miR-191-5p | ACA CTC CAG CTG GGC AAC GGA ATC CCA AA |
| dgl-miR-193a-5p | ACA CTC CAG CTG GGT GGG TCT TTG CGG GC |
| dgl-miR-193b-5p | ACA CTC CAG CTG GGC GGG GTT TTG AGG GC |
| dgl-miR-195-5p | ACA CTC CAG CTG GGT AGC AGC ACA GAA AT |
| dgl-miR-196a-5p | ACA CTC CAG CTG GGT AGG TAG TTT CAT GT |
| dgl-miR-199a-5p | ACA CTC CAG CTG GGC CCA GTG TTC AGA CT |
| dgl-miR-210-5p | ACA CTC CAG CTG GGA GCC ACT GCC CAC CG |
| dgl-miR-214-5p | ACA CTC CAG CTG GGT GCC TGT CTA CAC TT |
| dgl-miR-216a-5p | ACA CTC CAG CTG GGT AAT CTC AGC TGG CA |
| dgl-miR-218-5p | ACA CTC CAG CTG GGT TGT GCT TGA TCT AA |
| dgl-miR-219a-5p | ACA CTC CAG CTG GGT GAT TGT CCA AAC GC |
| dgl-miR-222-5p | ACA CTC CAG CTG GGC TCA GTA GCC AGT GT |
| dgl-miR-299-5p | ACA CTC CAG CTG GGT GGT TTA CCG TCC CA |
| dgl-miR-335-5p | ACA CTC CAG CTG GGT CAA GAG CAA TAA CG |
| dgl-miR-361-5p | ACA CTC CAG CTG GGT TAT CAG AAT CTC CA |
| dgl-miR-362-5p | ACA CTC CAG CTG GGA ATC CTT GGA ACC TA |
| dgl-miR-365b-5p | ACA CTC CAG CTG GGA GGG ACT TTC AGG GG |
| dgl-miR-375-5p | ACA CTC CAG CTG GGG CGA CGA GCC CCT CG |
| dgl-miR-377-5p | ACA CTC CAG CTG GGA GAG GTT GCC CTT GG |
| dgl-miR-379-5p | ACA CTC CAG CTG GGT GGT AGA CTA TGG AA |
| dgl-miR-381-5p | ACA CTC CAG CTG GGA GCG AGG TTG CCC TT |
| dgl-miR-409-5p | ACA CTC CAG CTG GGA GGT TAC CCG AGC AA |
| dgl-miR-411-5p | ACA CTC CAG CTG GGT AGT AGA CCG TAT AG |
| dgl-miR-425-5p | ACA CTC CAG CTG GGA ATG ACA CGA TCA CT |
| dgl-miR-429-5p | ACA CTC CAG CTG GGG TCT TAC CAG ACA CG |
| dgl-miR-452-5p | ACA CTC CAG CTG GGT GTT TGC AGA GGA AA |
| dgl-miR-483-5p | ACA CTC CAG CTG GGA AGA CGG GAG GAA AG |
| dgl-miR-485-5p | ACA CTC CAG CTG GGA GAG GCT GGC CGT GA |
| dgl-miR-491-5p | ACA CTC CAG CTG GGA GTG GGG AAC CCT TC |
| dgl-miR-500a-5p | ACA CTC CAG CTG GGT AAT CCT TGC TAC CT |
| dgl-miR-590-5p | ACA CTC CAG CTG GGG AGC TTA TTC ATA AA |
| dgl-miR-615-5p | ACA CTC CAG CTG GGG GGG GTC CCC GGT GC |
| dgl-miR-874-5p | ACA CTC CAG CTG GGC GGC CCC ACG CAC CA |
| dgl-miR-876-5p | ACA CTC CAG CTG GGT GGA TTT CTT TGT GA |
| U6 snRNA | ACA CTC CAG CTG GGG TGC TCG CTT CGG CAG C |
| Universal Primer | CTC ACA GTA CGT TGG TAT CCT TGT G |
| Stem-loop Adapter | CTC ACA GTA CGT TGG TAT CCT TGT GAT GTT CGA TGC CAT ATT GTA CTG TGA GTT TTT TTT TVN |

**Supplementary Table S2.** The relative expression levels of 85 miRNA species examined in liver and skeletal muscle of *D. gliroides*. MicroRNA relative expression was evaluated by qPCR of reverse-transcribed, polyadenylated transcripts. Data represent means of *n* = 4 biological replicates from different animals ± SEM. Relative expression of genes was calculated by standardizing against U6 snRNA expression. Control values were adjusted to 1 and the torpid values were expressed relative to the controls. Statistical testing used the Student's *t*-test; *significantly different from the corresponding control, *p* < 0.05.

| **MicroRNA** | **Liver** | | **Skeletal Muscle** | |
| --- | --- | --- | --- | --- |
|  | **Relative expression** | ***p*-value** | **Relative expression** | ***p*-value** |
| dgl-miR-let-7f-5p | 0.58 ± 0.08 | 0.034* | 1.45 ± 0.08 | 0.228 |
| dgl-miR-1a-5p | 0.32 ± 0.08 | 0.037* | 1.61 ± 0.08 | 0.002* |
| dgl-miR-1b-5p | 0.50 ± 0.07 | 0.001* | 1.65 ± 0.07 | 0.0006* |
| dgl-miR-7a-5p | 0.74 ± 0.13 | 0.142 | 0.93 ± 0.14 | 0.69 |
| dgl-miR-9-3-5p | 0.59 ± 0.04 | 0.07 | 1.02 ± 0.11 | 0.873 |
| dgl-miR-10b-5p | 0.57 ± 0.05 | 0.006* | 0.82 ± 0.14 | 0.306 |
| dgl-miR-15a-5p | 0.977 ± 0.09 | 0.862 | 1.51 ± 0.28 | 0.386 |
| dgl-miR-15b-5p | 0.906 ± 0.11 | 0.575 | 1.38 ± 0.3 | 0.455 |
| dgl-miR-16-3p | 0.79 ± 0.07 | 0.048* | 0.67 ± 0.11 | 0.049* |
| dgl-miR-17-5p | 0.84 ± 0.13 | 0.339 | 1.21 ± 0.27 | 0.575 |
| dgl-miR-18a-3p | 0.39 ± 0.03 | 0.012* | 1.01 ± 0.2 | 0.96 |
| dgl-miR-20a-5p | 0.40 ± 0.06 | 0.009* | 1.68 ± 0.3 | 0.113 |
| dgl-miR-21a-3p | 0.37 ± 0.03 | 0.028* | 0.72 ± 0.07 | 0.301 |
| dgl-miR-22-5p | 0.77 ± 0.04 | 0.032* | 0.58 ± 0.11 | 0.025* |
| dgl-miR-23a-5p | 0.44 ± 0.03 | 0.029* | 0.77 ± 0.06 | 0.17 |
| dgl-miR-26a-5p | 0.52 ± 0.11 | 0.110* | 1.53 ± 0.24 | 0.171 |
| dgl-miR-27a-5p | 0.45 ± 0.03 | 0.005* | 0.85 ± 0.13 | 0.323 |
| dgl-miR-28-5p | 0.98 ± 0.24 | 0.93 | 1.15 ± 0.2 | 0.592 |
| dgl-miR-29a-5p | 0.43 ± 0.03 | 0.005* | 2.18 ± 0.18 | 0.11 |
| dgl-miR-30a-5p | 0.86 ± 0.11 | 0.433 | 0.678 ± 0.13 | 0.298 |
| dgl-miR-30c-5p | 0.89 ± 0.10 | 0.423 | 0.62 ± 0.11 | 0.253 |
| dgl-miR-30d-5p | 1.25 ± 0.10 | 0.093 | 0.67 ± 0.13 | 0.313 |
| dgl-miR-30e-5p | 0.68 ± 0.13 | 0.081 | 0.68 ± 0.13 | 0.283 |
| dgl-miR-32-5p | 0.60 ± 0.1 | 0.071 | 1.24 ± 0.1 | 0.313 |
| dgl-miR-33a-5p | 0.62 ± 0.08 | 0.196 | 0.57 ± 0.11 | 0.021* |
| dgl-miR-34a-5p | 0.62 ± 0.04 | 0.014* | 0.71 ± 0.16 | 0.236 |
| dgl-miR-34c-5p | 0.50 ± 0.07 | 0.022* | 0.69 ± 0.3 | 0.382 |
| dgl-miR-96-5p | 0.97 ± 0.06 | 0.637 | 0.86 ± 0.08 | 0.184 |
| dgl-miR-99b-5p | 0.49 ± 0.04 | 0.006* | 1.32 ± 0.08 | 0.049* |
| dgl-miR-106b-5p | 0.50 ± 0.05 | 0.002* | 1.07 ± 0.11 | 0.69 |
| dgl-miR-124-3-5p | 0.72 ± 0.14 | 0.296 | 1.27 ± 0.1 | 0.35 |
| dgl-miR-125a-5p | 0.47 ± 0.07 | 0.001* | 1.5 ± 0.27 | 0.243 |
| dgl-miR-127-5p | 0.85 ± 0.07 | 0.315 | 0.81 ± 0.12 | 0.349 |
| dgl-miR-129-1-5p | 1.5 ± 0.4 | 0.301 | 1.22 ± 0.09 | 0.108 |
| dgl-miR-130b-5p | 0.52 ± 0.04 | 0.165 | 07 ± 0.10 | 0.12 |
| dgl-miR-132-5p | 0.82 ± 0.10 | 0.335 | 1.15 ± 0.22 | 0.551 |
| dgl-miR-133a-5p | 0.79 ± 0.11 | 0.313 | 1.00 ± 0.1 | 0.987 |
| dgl-miR-133b-5p | 0.61 ± 0.09 | 0.127 | 0.90 ± 0.20 | 0.689 |
| dgl-miR-134-5p | 0.45 ± 0.04 | 0.038* | 0.937 ± 0.053 | 0.345 |
| dgl-miR-137-5p | 0.53 ± 0.03 | 0.0004* | 0.74 ± 0.15 | 0.263 |
| dgl-miR-139-5p | 0.59 ± 0.07 | 0.04* | 2.52 ± 0.28 | 0.011* |
| dgl-miR-142b-5p | 0.71 ± 0.07 | 0.021* | 1.02 ± 0.16 | 0.926 |
| dgl-miR-144-5p | 1.02 ± 0.32 | 0.96 | 1.36 ± 0.22 | 0.208 |
| dgl-miR-145a-5p | 0.42 ± 0.03 | 0.047* | 1.26 ± 0.2 | 0.422 |
| dgl-miR-147-5p | 0.89 ± 0.16 | 0.679 | 0.85 ± 0.08 | 0.425 |
| dgl-miR-152-5p | 0.57 ± 0.03 | 0.021* | 0.68 ± 0.14 | 0.12 |
| dgl-miR-181a-5p | 0.49 ± 0.06 | 0.013* | 1.05 ± 0.22 | 0.872 |
| dgl-miR-181a-3p | 1.38 ± 0.6 | 0.613 | 2.19 ± 0.37 | 0.047* |
| dgl-miR-185-5p | 0.36 ± 0.03 | 0.026* | 0.63 ± 0.07 | 0.006* |
| dgl-miR-186-5p | 0.36 ± 0.03 | 0.094 | 1.02 ± 0.1 | 0.915 |
| dgl-miR-190a-5p | 0.77 ± 0.11 | 0.133 | 1.49 ± 0.10 | 0.022* |
| dgl-miR-191-5p | 0.75 ± 0.03 | 0.014* | 1.01 ± 0.08 | 0.964 |
| dgl-miR-193a-5p | 0.48 ± 0.10 | 0.085 | 1.19 ± 0.16 | 0.404 |
| dgl-miR-193b-5p | 0.52 ± 0.07 | 0.015* | 0.81 ± 0.14 | 0.266 |
| dgl-miR-195-5p | 0.85 ± 0.12 | 0.449 | 1.23 ± 0.37 | 0.688 |
| dgl-miR-196a-5p | 0.54 ± 0.03 | 0.026* | 1.37 ± 0.31 | 0.448 |
| dgl-miR-199a-5p | 0.42 ± 0.04 | 0.086 | 1.15 ± 0.36 | 0.782 |
| dgl-miR-210-5p | 1.80 ± 0.82 | 0.408 | 2.64 ± 0.62 | 0.071 |
| dgl-miR-214-5p | 0.56 ± 0.07 | 0.006* | 1.24 ± 0.07 | 0.105 |
| dgl-miR-216a-5p | 1.57 ± 0.25 | 0.103 | 0.97 ± 0.071 | 0.781 |
| dgl-miR-218-5p | 0.85 ± 0.14 | 0.446 | 0.93 ± 0.16 | 0.68 |
| dgl-miR-219a-5p | 0.49 ± 0.07 | 0.028* | 0.77 ± 0.18 | 0.355 |
| dgl-miR-222-5p | 0.88 ± 0.06 | 0.315 | 0.58 ± 0.14 | 0.355 |
| dgl-miR-299-5p | 0.51 ± 0.11 | 0.145 | 0.73 ± 0.26 | 0.509 |
| dgl-miR-335-5p | 0.47 ± 0.06 | 0.019* | 1.05 ± 0.17 | 0.8 |
| dgl-miR-361-5p | 0.96 ± 0.22 | 0.897 | 0.66 ± 0.12 | 0.054 |
| dgl-miR-362-5p | 1.09 ± 0.19 | 0.704 | 0.75 ± 0.13 | 0.337 |
| dgl-miR-365b-5p | 0.50 ± 0.05 | 0.173 | 0.78 ± 0.07 | 0.258 |
| dgl-miR-375-5p | 1.69 ± 0.03 | 0.1 | 1.13 ± 0.11 | 0.611 |
| dgl-miR-377-5p | 0.47 ± 0.05 | 0.084 | 1.24 ± 0.14 | 0.205 |
| dgl-miR-379-5p | 0.63 ± 0.05 | 0.103 | 0.99 ± 0.18 | 0.973 |
| dgl-miR-381-5p | 0.49 ± 0.03 | 0.040* | 1.19 ± 0.09 | 0.143 |
| dgl-miR-409-5p | 0.89 ± 0.1 | 0.81 | 0.85 ± 0.13 | 0.596 |
| dgl-miR-411-5p | 0.88 ± 0.11 | 0.48 | 1.19 ± 0.05 | 0.355 |
| dgl-miR-425-5p | 0.45 ± 0.03 | 0.001* | 1.26 ± 0.18 | 0.24 |
| dgl-miR-429-5p | 0.75 ± 0.07 | 0.108 | 1.41 ± 0.16 | 0.08 |
| dgl-miR-452-5p | 0.83 ± 0.06 | 0.325 | 1.21 ± 0.13 | 0.214 |
| dgl-miR-483-5p | 1.34 ± 0.48 | 0.54 | 1.91 ± 0.21 | 0.016* |
| dgl-miR-485-5p | 1.21 ± 0.42 | 0.672 | 0.72 ± 0.13 | 0.173 |
| dgl-miR-491-5p | 1.02 ± 0.11 | 0.887 | 1.05 ± 0.06 | 0.801 |
| dgl-miR-500a-5p | 0.83 ± 0.22 | 0.495 | 0.9 ± 0.09 | 0.604 |
| dgl-miR-590-5p | 0.71 ± 0.3 | 0.464 | 0.99 ± 0.19 | 0.962 |
| dgl-miR-615-5p | 1.8 ± 0.52 | 0.257 | 0.86 ± 0.125 | 0.498 |
| dgl-miR-874-5p | 0.82 ± 0.26 | 0.56 | 1.34 ± 0.26 | 0.407 |
| dgl-miR-876-5p | 0.28 ± 0.06 | 0.021* | 0.85 ± 0.05 | 0.377 |
